# Active Surveillance Program to Increase Awareness on Invasive Fungal Diseases: the French RESSIF Network (2012 to 2018)

**DOI:** 10.1128/mbio.00920-22

**Published:** 2022-05-02

**Authors:** Stéphane Bretagne, Karine Sitbon, Marie Desnos-Ollivier, Dea Garcia-Hermoso, Valérie Letscher-Bru, Sophie Cassaing, Laurence Millon, Florent Morio, Jean-Pierre Gangneux, Lilia Hasseine, Loïc Favennec, Estelle Cateau, Eric Bailly, Maxime Moniot, Julie Bonhomme, Nicole Desbois-Nogard, Taieb Chouaki, André Paugam, Bernard Bouteille, Marc Pihet, Frédéric Dalle, Odile Eloy, Milène Sasso, Magalie Demar, Patricia Mariani-Kurkdjian, Vincent Robert, Olivier Lortholary, Françoise Dromer, the French Mycoses Study Group

**Affiliations:** a Institut Pasteur, Université Paris Cité, CNRS, Mycologie Moléculaire, Centre National de Référence Mycoses Invasives et Antifongiques, UMR 2000, Paris, France; b Laboratoire de Parasitologie-Mycologie, Hôpital Saint Louis, Assistance Publique-Hôpitaux De Paris (AP-HP), Paris, France; c Laboratory of Parasitology and Medical Mycology, Strasbourg University Hospital, Institute of Parasitology and Tropical Diseases, UR7292 Dynamics of Host-Pathogen Interactions, Federation of Translational Medicine, University of Strasbourg, Strasbourg, France; d Sophie Cassaing: Department of Parasitology and Mycology, CHU Toulouse, Restore Institute, Toulouse, France; e Laboratoire de Parasitologie-Mycologie, Centre Hospitalier Universitaire de Besançon, UMR 6249 CNRS Chrono-Environnement, University Bourgogne Franche-Comté, Besançon, France; f Nantes Université, CHU Nantes, Cibles et Médicaments des Infections et du Cancer, IICiMed, UR 1155, Nantes, France; g Univ Rennes, CHU, INSERM, Irset: Institut de Recherche en Santé, Environnement et Travail, UMR_S 1085, Rennes, France; h Laboratoire de Parasitologie-Mycologie, Centre Hospitalier Universitaire de Nice, Nice, France; i EA 7510, Centre Hospitalier UC. Nicolle, Rouen, France; j Laboratoire de Mycologie, CHU de Poitiers, UMR CNRS 7267, Poitiers, France; k Laboratoire de Parasitologie-Mycologie-Médecine Tropicale, CHRU Tours, France; l Service de Parasitologie-Mycologie, CHU Clermont-Ferrand, 3iHP, Clermont-Ferrand, France; m Department of Microbiology, University Hospital of Caen, ToxEMAC-ABTE, Unicaen Normandie University, Caen, France; n Laboratoire de Parasitologie-Mycologie, CHU de la Martinique, Fort de France, France; o Mycologie-Parasitologie, CHU d'Amiens, Amiens, France; p Laboratoire de Mycologie, Hôpital Cochin, Paris, France; q Department of Parasitology and Mycology, University Hospital, Limoges, France; r Laboratoire de Parasitologie-Mycologie, CHU d’Angers, University Angers, University Brest, GEIHP, SFR ICAT, Angers, France; s University Bourgogne Franche-Comté, Agrosup Dijon, UMR PAM A 02.102, Dijon, France; t Laboratoire de Parasitologie-Mycologie, Plateforme de Biologie Hospitalo-Universitaire Gérard Mack, Dijon, France; u Centre Hospitalier de Versailles, Le Chesnay, France; v Laboratoire de Parasitologie-Mycologie, Centre Hospitalier Universitaire de Nîmes, Université de Montpellier, CNRS, IRD, UMR MiVEGEC, Montpellier, France; w Laboratoire Hospitalo-Universitaire de Parasito-Mycologie, centre hospitalier de Cayenne Guyane, Cayenne, France; x CHU Robert Debré, Paris, France; y Bioinformatics Group, Westerdijk Fungal Biodiversity Institute, Utrecht, The Netherlands; z Centre for Infectious Diseases and Tropical Medicine, Hôpital Universitaire Necker-Enfants Malades, Assistance Publique, Hôpitaux de Paris, Université Paris Cité, Paris, France; CDC

**Keywords:** aspergillosis, mucormycosis, pneumocytosis, candidemia, epidemiology, invasive fungal infections

## Abstract

The French National Reference Center for Invasive Mycoses and Antifungals leads an active and sustained nationwide surveillance program on probable and proven invasive fungal diseases (IFDs) to determine their epidemiology in France. Between 2012 and 2018, a total of 10,886 IFDs were recorded. The incidence increased slightly over time (2.16 to 2.36/10,000 hospitalization days, *P* = 0.0562) in relation with an increase of fungemia incidence (1.03 to 1.19/10,000, *P* = 0.0023), while that of other IFDs remained stable. The proportion of ≥65-year-old patients increased from 38.4% to 45.3% (*P* < 0.0001). Yeast fungemia (*n* = 5,444) was due mainly to Candida albicans (55.6%) with stable proportions of species over time. Echinocandins became the main drug prescribed (46.7% to 61.8%), but global mortality rate remained unchanged (36.3% at 1 month). Pneumocystis jirovecii pneumonia (*n* = 2,106) was diagnosed mostly in HIV-negative patients (80.7%) with a significantly higher mortality than in HIV-positive patients (21.9% versus 5.4% at 1 month, *P* < 0.0001). Invasive aspergillosis (*n* = 1,661) and mucormycosis (*n* = 314) were diagnosed mostly in hematology (>60% of the cases) with a global mortality rate of 42.5% and 59.3%, respectively, at 3 months and significant changes in diagnosis procedure over time. More concurrent infections were also diagnosed over time (from 5.4% to 9.4% for mold IFDs, *P* = 0.0115). In conclusion, we observed an aging of patients with IFD with a significant increase in incidence only for yeast fungemia, a trend toward more concurrent infections, which raises diagnostic and therapeutic issues. Overall, global survival associated with IFDs has not improved despite updated guidelines and new diagnostic tools.

## INTRODUCTION

Invasive fungal diseases (IFDs) are mainly opportunistic diseases, and their burden is difficult to assess in the absence of national surveillance programs in most countries. Global estimates by the Leading International Fungal Education (LIFE) initiative are available based on publications from reference laboratories (1). The LIFE initiative raises awareness on the epidemiology of IFDs and on the source of information used (1). Differences are expected since the prevalence of opportunistic diseases depends on global health care systems without mentioning the impact of reporting means, diagnostic tools, and geographical location. The hospital network and the distribution of medical specialties are uneven across a country, resulting in differences in the geographical breakdown of high-risk patients and therefore IFDs. A major hurdle for accurate data on IFDs is also their diagnosis that requires multidisciplinary teams analyzing clinical, imaging, and microbiological data (2).

Most surveys focus on yeast fungemia, which is the easiest IFD to record. Many registries focus on specific populations, such as solid organ transplant recipients (3, 4). Networks such as SENTRY (5) or CHIF-NET (6) provide microbiological data and others such as FungiScope (7) add clinical information. Several national reference centers (NRCs) on fungal infections exist in Europe with predominant missions of microbiological expertise and sometimes service for diagnosis or management advice. Thus, the Danish NRC conducts a surveillance of candidemia (8) and azole-resistant Aspergillus fumigatus (9). Others survey candidemia through nationwide surveillance (10) or multicenter surveys in Germany, Belgium, Spain, or Bulgaria (11). Specific investigations are punctually led by the European Centre for Disease Prevention and Control (12).

Many studies rely on passive notification (13, 14), but other sources of information are available. Bitar et al. reported an overall incidence of 5.9/100,000 cases/year based on the French National Hospital Discharge Database (2001 to 2010), with a global mortality rate of 27.6% (15). More recently, Webb et al. analyzed the Intermountain Healthcare Enterprise Data Warehouse (2006 to 2015) and described an incidence of IFDs (excluding Pneumocystis jirovecii pneumonia [PJP]) of 27.2 cases/100,000 patients per year with an all-cause mortality of 17.0% at 42 days (16). Differences may be related to the populations studied and the sample size (35,876 versus 3,374 cases). However, both studies acknowledged that electronic data extraction is likely to result in some inaccuracies, even after extensive manual validation (15, 16). Indeed, misdiagnosis and incorrect coding can result in moderate performances at least in specific populations (17). Furthermore, some diagnoses require the identification of the causal agent (16). The advent of new diagnostic tools and the grouping of laboratories in large platforms may also substantially impact temporal trends.

Active and sustained surveillance systems based on local multidisciplinary teams should overcome some limitations, but such networks are rare. In France, the assignments of NRCs supervised by Santé Publique France (SPF) include expertise and surveillance on specific microorganisms and contribution to outbreak investigations. The NRC for Invasive Mycoses and Antifungals (NRCMA) deals with all pathogenic fungi and is also in charge of surveying all IFDs. Up to 2012, it relied on passive surveillance except for yeast fungemia for which the active surveillance YEASTS program was implemented (18–21). In 2012, a network called RESeau de Surveillance des Infections Fongiques (RESSIF) was launched to actively survey all proven and probable IFDs and underlying conditions and to characterize the isolates of uncommon species and/or unusual phenotypes at the NRCMA. RESSIF is still ongoing. Our purpose here is to describe its organization and the IFD burden in France between 2012 and 2018. It is also to officially launch the open access database Institut Pasteur FungiBank (https://fungibank.pasteur.fr/) recording data (antifungal susceptibility profiles, sequences, and major clinical settings) associated with the uncommon species responsible for IFDs in France.

## RESULTS

### Global picture of IFDs in France.

**FIG 1:**
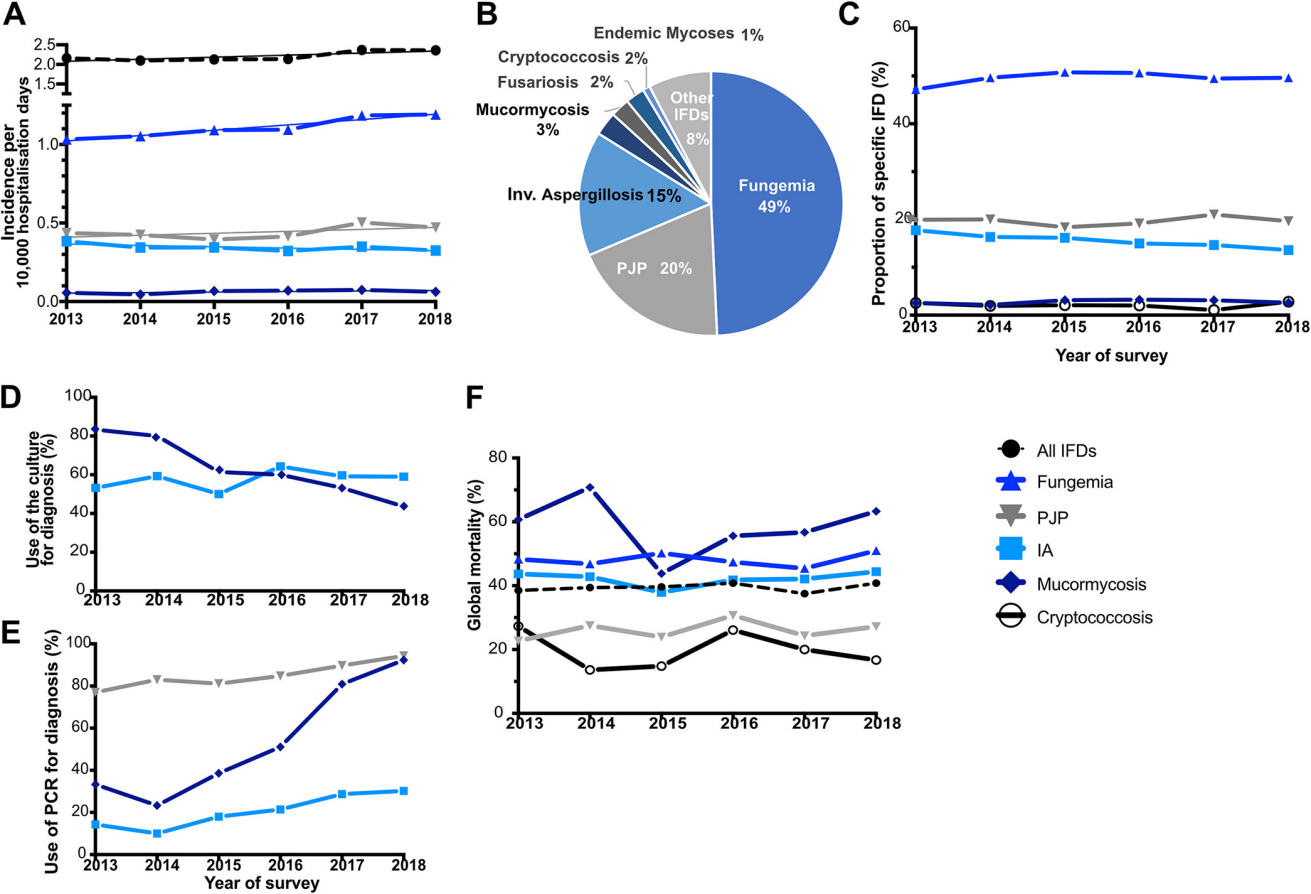
Invasive fungal diseases (IFDs) and related underlying conditions and their evolution (RESSIF network, France, 2012 to 2018). (A) Evolution of the incidence of the major IFDs/10,000 hospitalization days in the 21 centers participating sustainably to the RESSIF network between 2013 and 2018. A linear regression was performed for all IFDs (*P* = 0.0562), fungemia (*P* = 0.0023), pneumocystosis (PJP; *P* = 0.2274), invasive aspergillosis (IA; *P* = 0.1029), and mucormycosis (*P* = 0.1886). (B) Proportion of the major IFDs among the 10,886 episodes recorded through the RESSIF network (2012 to 2018). (C to F) Trends in the 21 centers participating sustainably to the RESSIF network between 2013 and 2018 (chi-squared test for trends). (C) Proportion of fungemia (*P* = 0.3022), PJP (*P* = 0.7649), mucormycosis (*P* = 0.3801), cryptococcosis (*P* = 0.6689), and IA (*P* = 0.0007) recorded. (D) Culture as a tool for the diagnosis of IA (*P* = 0.1524) and mucormycosis (*P* < 0.0001). (E) PCR-based methods as a tool for the diagnosis of PJP, IA, and mucormycosis (*P* < 0.0001). (F) Global mortality rate at 3 months for all and selected IFDs (not significant).

Over the 7 years of the study, a total of 10,886 IFDs were recorded in 10,154 patients. Overall, the global incidence of IFD was 2.21/10,000 hospitalization days with a slight increase over time (2.16 to 2.36, *P* = 0.0562) in relation with an increased incidence of fungemia (1.03 to 1.19/10,000, *P* = 0.0023), while that of other IFDs remained stable (Fig. 1A). There was a 6% decrease in the number of hospitalization days from 2014 until 2018 in the participating centers. Overall, there was a significant increase in concurrent infections diagnosed when considering all IFDs (3.4% to 5.6%, *P* = 0.0092) or only mold IFDs (from 5.4% to 9.4%, *P* = 0.0115).

The collaborative center NRCMAs (CC-NRCMAs) sent >90% of uncommon species (2,601 isolates). Discrepancies in the final identification were due essentially to changes in the current nomenclature. They concerned the genera (*n* = 102, 3.9%) or species within the correct genera (*n* = 370, 14.2%) and mainly for molds (82/102 and 328/370, respectively).

### Characteristics of the patients and trends over time.

**FIG 2:**
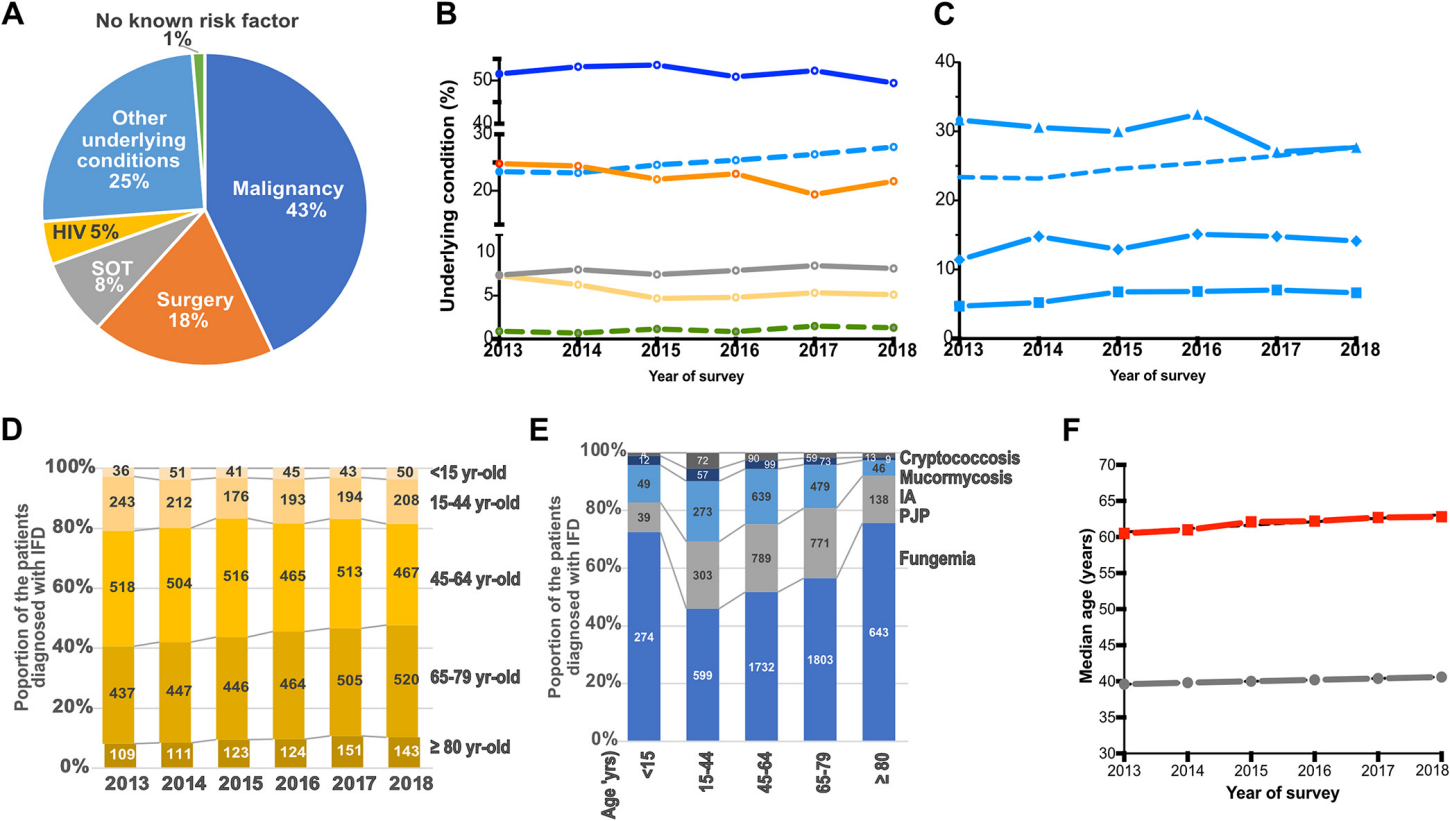
Underlying conditions associated with the diagnosis of invasive fungal diseases in 10,154 patients (RESSIF network, France, 2012 to 2018). (A) Proportion of the major exclusive underlying conditions in the 10,154 patients. (B) Trends in the evolution of the following major underlying conditions in the 21 centers participating sustainably to the RESSIF network between 2013 and 2018: malignancy (blue solid line; *P* = 0.1583), solid organ transplantation (gray line; *P* = 0.3496), recent surgery (orange line; *P* = 0.0017), HIV infection (yellow line; *P* = 0.0097), other risk factors (dotted pale-blue line; *P* = 0.0009), and no known risk factor (green line; *P* = 0.0720) (chi-squared test for trends). (C) Trends in the evolution of associated risk factors, as follows: diabetes (diamond; *P* = 0.0443), stay in intensive care unit (triangle; *P* = 0.0061), autoimmune disorders (square; *P* = 0.0055), and other risk factor (same dotted line shown in B) (chi-squared test for trends). (D) Histograms showing the evolution of age over time in the 5 age categories (80 years old and over, 65 to 79 years old, 45 to 64 years old, 15 to 44 years old, and <15 years old old) in a range of yellow getting darker with age. The figures correspond to the number of cases. The proportion of older patients increased significantly (from 8.1% to 10.6% for ≥80 years old, *P* = 0.0026; and from 30.3% to 34.7% for those between 65 and <80 years old, *P* = 0.0045) in the 21 centers participating sustainably to the RESSIF network between 2013 and 2018. (E) Histograms showing the relative distribution of the major IFDs, fungemia (blue), PJP (pale gray), IA (pale blue), mucormycosis (dark blue), and cryptococcosis (dark gray) in the 241 children <15 years old, 1,001 patients 15 to 44 years old, 2,687 patients 45 to 64 years old, 2,591 patients 65 to 79 years old, and 710 patients ≥80 years old. The figures correspond to the number of cases. (F) Evolution of the median age of the French population (gray line; data available online at https://www.insee.fr/fr/statistiques/2381476) and of the patients diagnosed with IFDs in the 21 centers (red line, linear regression; *P* = 0.0027).

Most of the patients were adults (9,738/10,154, 95.9%) with a median age of 61.4 years (interquartile range [IQR], 22.7) (Table 1 and 2).IFDs were more common in men (male:female [M:F] ratio, 1.7:1 in adults; 1.2:1 in children; *P* = 0.002). The main exclusive underlying conditions were malignancies (*n* = 4,384, 43.2%), surgery (*n* = 1,854, 18.3%), solid organ transplantation (SOT; *n* = 798, 7.9%), HIV infection (*n* = 454, 4.5%), others (*n* = 2,539, 25.0%), and none (*n* = 125, 1.2%) (Fig. 2A). Overall, 12.5% of the patients (*n* = 1,271) had multiple underlying conditions (see Table S1 in the supplemental material). Thus, 5,140 patients had malignancies (including 2,634 [51.2%] hematological malignancies [HM], 2,364 [46.0%] solid tumor, and 142 [2.8%] both), 2,264 recent surgeries, 873 SOT, and 638 HIV infection. When looking at single conditions over time (Fig. 2B), there was a decrease in the proportion of patients with recent surgery (from 24.8% to 21.7%, *P* = 0.0017) or HIV infection (from 7.3% to 5.1%, *P* = 0.0097) and an increase in those with other conditions (from 23.4% to 27.7%, *P* = 0.0009), related to an increase in patients with diabetes (11.4 to 14.1%, *P* = 0.0443) or autoimmune diseases (4.7% to 6.6%, *P* = 0.0055), while the proportion of patients in the intensive care unit (ICU) decreased (31.7% to 27.7%, *P* = 0.0069) (Fig. 2C). The proportion of the older patients (≥65 years old) increased from 38.4% to 45.3% (*P* < 0.0001) (Fig. 2D and E). The median age in years of patients diagnosed with IFDs increased by more than 2 years (60.5 to 62.8), while, at the same time, that of the French population increased by 1 year (39.6 to 40.6) (Fig. 2F). More diabetes and autoimmune disorders were reported in the 4,152 patients over 65 years old (*n* = 820 [19.8%] and *n* = 321 [7.7%]) than in the younger ones (*n* = 624 [10.4%] and *n* = 306 [5.1%]) (*P* < 0.0001).

**TABLE 1:** Characteristics of the 10,886 episodes in 10,154 patients diagnosed with IFDs recorded through the RESSIF network^*a*^

Parameter	Results^*b*^ by IFDs									Total results^*b*^	*P* value
	Fungemia^*c*^ (*n* = 5,363)	Pneumocystosis (*n* = 2,106)	Invasive aspergillosis (*n* = 1,661)	Mucormycosis (*n* = 314)	Fusariosis by type		Cryptococcosis^*d*^ (*n* = 255)	Endemic mycoses (*n* = 96)	Other IFDs (*n* = 837)		
					Deep (*n* = 92)	Ocular (*n* = 162)					
Characteristics of the patients											
Male sex	3,106/5,056 (61.4)	1,322/2,038 (64.9)	933/1,481 (63.0)	167/253 (66.0)	104/237 (43.9)	160/240 (66.7)	160/240 (66.7)	64/90 (71.1)	482/759 (63.5)	6,338/1,0154 (62.4)	<0.0001
Median age (years [IQR^*e*^])	63.4 (22.8)	61.95 (20.9)	59.2 (20.7)	57.5 (24.1)	55.15 (26.65)	36.9 (25)	52.35 (26)	48.4 (24.4)	60 (24.9)	61.4 (22.7)	<0.0001
Main underlying risk factor											
Malignancy	1,908/5,056 (37.7)	971/2,038 (47.6)	1,058/1,481 (71.4)	160/253 (63.2)	44/76 (57.9)	1/161 (0.6)	66/240 (27.5)	5/90 (5.6)	171/759 (22.5)	4,384/10,154 (43.2)	
Malignancy, including hematological malignancy	782/1,908 (41.0)	574/971 (59.1)	968/1,058 (91.5)	155/160 (96.9)	42/44 (95.5)		50/66 (75.8)	5/5 (100.0)	105/171 (61.4)	2,682/4,384 (61.2)	
SOT	162/5,056 (3.2)	310/2,038 (15.2)	163/1,481 (11.0)	17/253 (6.7)	2/76 (2.6)		44/240 (18.3)	4/90 (4.4)	96/759 (12.6)	798/10,154 (7.9)	
Recent surgery	1,610/5,056 (31.8)	5/2,038 (0.2)	17/1,481 (1.1)	5/253 (2.0)	2/76 (2.6)	2/161 (1.2)			213/759 (28.1)	1,854/10,154 (18.3)	
HIV infection	11/5,056 (0.2)	334/2,038 (16.4)	2/1,481 (0.1)				63/240 (26.3)	39/90 (43.3)	5/759 (0.7)	454/10,154 (4.5)	
Other risk factor	1,351/5,056 (26.7)	405/2,038 (19.9)	224/1,481 (15.1)	68/253 (26.9)	26/76 (34.2)	149/161 (92.5)	51/240 (21.3)	17/90 (18.9)	248/759 (32.7)	2,539/10,154 (25.0)	
No known underlying condition	14/5,056 (0.3)	13/2,038 (0.6)	17/1,481 (1.1)	3/253 (1.2)	2/76 (2.6)	9/161 (5.6)	16/240 (6.7)	25/90 (27.8)	26/759 (3.4)	125/10,154 (1.2)	
Characteristics of the isolates											
Intensive care unit	1,924/5,363 (35.9)	564/2,106 (26.8)	536/1,661 (32.3)	100/314 (31.8)	27/92 (29.3)	1/162 (0.6)	42/255 (16.5)	10/96 (10.4)	163/837 (19.5)	3,367/10,886 (30.9)	<0.0001
Type of IFD											<0.0001
Proven	5,363/5,363 (100.0)	1,172/2,106 (55.7)	261/1,661 (15.7)	160/314 (51.0)	68/92 (73.9)	73/162 (45.1)	243/255 (95.3)	93/96 (96.9)	698/837 (83.4)	8,130/10,886 (74.7)	
Probable		645/2,106 (30.6)	1,387/1,661 (83.5)	100/314 (31.8)	24/92 (26.1)	89/162 (54.9)	12/255 (4.7)	1/96 (1.0)	136/837 (16.2)	2,394/10,886 (22.0)	
PCR only		289/2,106 (13.7)	13/1,661 (0.8)	54/314 (17.2)				2/96 (2.1)	3/837 (0.4%)	362/10,886 (3.3%)	
Means of diagnosis											
Positive culture	5,363/5,363 (100.0)		952/1,661 (57.3)	203/314 (64.6)	91/92 (98.9)	162/62 (261.3)	232/255 (91.0)	77/96 (80.2)	782/837 (93.4)	7,861/10,886 (72.2)	<0.0001
Fungal elements in fluids/tissues	1,715/5,363 (32.0)	947/2,106 (45.0)	579/1,661 (34.9)	193/314 (61.5)	49/92 (53.3)	68/62 (109.7)	151/255 (59.2)	72/96 (75.0)	378/837 (45.2)	4,152/10,886 (38.1)	<0.0001
Positive antigen detection	253/5,363 (4.7)	189/2,106 (9.0)	1,225/1,661 (73.8)	23/314 (7.3)	12/92 (13.0)		179/255 (70.2)	12/96 (12.5)	76/837 (9.1)	1,969/10,886 (18.1)	<0.0001
Positive PCR test	9/5,363 (0.2)	1,786/2,106 (84.8)	314/1,661 (18.9)	168/314 (53.5)	5/92 (5.4)		7/255 (2.7)	24/96 (25.0)	46/837 (5.5)	2,359/10,886 (21.7)	<0.0001
Initial antifungal treatment											
Caspofungin	2,366/4,592 (51.5)	3/1,976 (0.2)	51/1,554 (3.3)	3/283 (1.1)	2/83 (2.4)			1/82 (1.2)	169/729 (23.2)	2,595/9,657 (26.9)	
Fluconazole	1,424/4,592 (31.0)	5/1,976 (0.3)	4/1,554 (0.3)	0/283 (0.0)	2/83 (2.4)		50/224 (22.3)	2/82 (2.4)	189/729 (25.9)	1,676/9,657 (17.4)	
Voriconazole	100/4,592 (2.2)	7/1,976 (0.4)	1,046/1,554 (67.3)	8/283 (2.8)	29/83 (34.9)	38/134 (28.4)	1/224 (0.4)	4/82 (4.9)	119/729 (16.3)	1,352/9,657 (14.0)	
Liposomal amphotericin B	150/4,592 (3.3)	4/1,976 (0.2)	247/1,554 (15.9)	176/283 (62.2)	25/83 (30.1)	6/134 (4.5)	15/224 (6.7)	32/82 (39.0)	58/729 (8.0)	713/9,657 (7.4)	
Cotrimoxazole		1,813/1,976 (91.8)								1,813/9,657 (18.8)	
Other drugs or combinations	552/4,592 (12.0)	144/1,976 (7.3)	206/1,554 (13.3)	96/283 (33.9)	25/83 (30.1)	90/134 (67.2)	158/224 (70.5)	43/82 (52.4)	194/729 (26.6)	1,508/9,657 (15.6)	
Global mortality at 3 mo	2,003/4,204 (47.6)	461/1,777 (25.9)	562/1,385 (40.6)	139/241 (57.)	24/69 (34.8%)	0/93 (0.0)	41/213 (19.2)	15/73 (20.5)	118/613 (19.2)	3,363/8,668 (38.8)	<0.0001

a France, 2012 to 2018.

b Results are *n*/*n* total^*b*^ (%) except where otherwise noted. *n* total, total no. of patients for whom the corresponding IFD was the first episode recorded.

c Excluding blood culture positive with Cryptococcus neoformans (*n *= 97) that are recorded among the cryptococcosis cases.

d Only 3 cases due to Candida gattii were recorded among 3 HIV-negative patients.

e IQR, interquartile range.

**TABLE 2:** Comparison of the main underlying conditions in 10,154 patients diagnosed with 10,886 invasive fungal diseases recorded through RESSIF network^*a*^

Parameter	Results^*b*^ by IFD type						*P* value
	Malignancies (*n* = 4,745)	Surgery (*n* = 1,922)	SOT (*n* = 881)	HIV infection (*n* = 482)	Other conditions (*n* = 2730)	No known risk factor (*n* = 126)	
Characteristics of the patients							
Male sex	2,707/4,384 (61.7)	1,148/1,854 (61.9)	505/798 (63.3)	334/454 (73.6)	1,559/2,539 (61.4)	85/125 (68.0)	<0.0001
Median age (IQR)	62.6 (18.5)	65.1 (22.5)	59.4 (16.5)	46.45 (18.5)	62.6 (16.4)	59.4 (31.7)	<0.0001
Additional underlying conditions							
Diabetes	453/4,384 (10.3)	326/1,854 (17.6)	202/798 (25.3)	2/454 (0.4)	461/2,539 (18.2)		<0.0001
Cirrhosis	69/4,384 (1.6)	78/1,854 (4.2)	60/798 (7.5)	1/454 (0.2)	216/2,539 (8.5)		<0.0001
Autoimmune diseases	126/4,384 (2.9)	63/1,854 (3.4)	35/798 (4.4)		403/2,539 (15.9)		<0.0001
Corticosteroid therapy (>0.3 mg/kg, >1 mo)	1087/4,384 (24.8)	99/1,854 (5.3)	361/798 (45.2)	2/454 (0.4)	421/2,539 (16.6)		<0.0001
Characteristics of the episodes							
Hospitalization in ICU	1,152/4,745 (24.3)	774/1,922 (40.3)	289/881 (32.8)	110/482 (22.8)	1,042/2,730 (38.2)		<0.0001
Prior exposure to antifungals	1,052/4,574 (23.0)	170/1,871 (9.1)	153/850 (18.0)	43/467 (9.2)	271/2,644 (10.2)		<0.0001
Multiple episodes (simultaneous or subsequent)	326/4,384 (7.4)	74/1,854 (4.0)	64/798 (8.0)	33/454 (7.3)	145/2,539 (5.7)	1/125 (0.8)	<0.0001
Concurrent infections (same or different body sites)	273/4,745 (5.8)	17/1,922 (0.9)	49/881 (5.6)	15/482 (3.1)	114/2,730 (4.2)	2/126 (1.6)	<0.0001
Recurrence	96/4,745 (2.0)	24/1,922 (1.2)	21/881 (2.4)	19/482 (3.9)	75/2,730 (2.7)	2/126 (1.6)	0.0001
Median delay reporting in mo (IQR)	4.3 (10.2)	3.7 (8.5)	4.7 (10.9)	4.2 (11.7)	3.9 (9.1)	3.7 (7.3)	<0.0001
Classification of the IFD							
Proven	2,998/4,745 (63.2)	1,887/1,922 (98.2)	583/881 (66.2)	387/482 (80.3)	2,190/2,730 (80.2)	95/126 (75.4)	<0.0001
Probable	1,553/4,745 (32.7)	35/1,922 (1.8)	259/881 (29.4)	68/482 (14.1)	455/2,730 (16.7)	24/126 (19.0)	
PCR only	204/4,745 (4.3)		39/881 (4.4)	27/482 (5.6)	85/2,730 (3.1)	7/126 (5.6)	
Means of diagnosis							
Positive culture	2,957/4,745 (62.3)	1,915/1,922 (99.6)	510/881 (57.9)	131/482 (27.2)	2,248/2,730 (82.3)	100/126 (79.4)	<0.0001
Fungal elements in fluids/tissues	1,538/4,745 (32.4)	628/1,922 (32.7)	456/881 (51.8)	356/482 (73.9)	1,098/2,730 (40.2)	76/126 (60.3)	<0.0001
Positive antigen detection (serum ± respiratory samples)	1,254/4,745 (26.4)	94/1,922 (4.9)	216/881 (24.5)	98/482 (20.3)	290/2,730 (10.6)	17/126 (13.5)	<0.0001
Positive PCR test	1,236/4,745 (26.0)	12/1,922 (0.6)	325/881 (36.9)	262/482 (54.4)	498/2,730 (18.2)	26/126 (20.6)	<0.0001
Global mortality at 3 mo	1,783/3,843 (46.4)	577/1,523 (37.9)	184/740 (24.9)	35/390 (9.0)	776/2,083 (37.3)	8/89 (9.0)	<0.0001

a France, 2012 to 2018.

b Results are *n*/*n* total (%) unless otherwise stated.

10.1128/mbio.00920-22.2 TABLE S1 Underlying conditions in 10,154 patients diagnosed with invasive fungal diseases in France (RESSIF, 2013 to 2018). Download Table S1, PDF file, 0.02 MB. Copyright © 2022 Bretagne et al. 2022 Bretagne et al. https://creativecommons.org/licenses/by/4.0/ This content is distributed under the terms of the Creative Commons Attribution 4.0 International license.

Overall, 6.3% (*n* = 643) of the patients were diagnosed with several IFDs (median interval of 23 days [range 0 to 2,431]). Multiple infections were more frequent in patients with pre-exposure to antifungal drugs (146/1,371 [10.7%] versus 476/8,443 [5.6%], *P* < 0.0001).

Among the 10,886 episodes of IFDs, fungemia accounted for almost one-half of them (*n* = 5,363, 49.3%), followed by PJP (*n* = 2,106, 19.4%), invasive aspergillosis (IA; *n* = 1,661, 15.3%), mucormycosis (*n* = 314, 2.9%), cryptococcosis (*n* = 255, 2.3%), fusariosis (*n* = 254, 2.3%), endemic mycoses (*n* = 96, 0.9%), and other IFDs (*n* = 837, 7.7%) (Fig. 1B). Concurrent infections, mostly IA/mucormycosis (*n* = 109), IA/PJP (*n* = 104), and IA/fungemia (*n* = 93), were diagnosed in 4.3% (*n* = 470) of the cases. Mixed species were recorded in 342 cases with no change over time (data not shown) and identified mostly during fungemia (4.4%) and IA (2.8%). There was a significant decrease in the proportion of IA recorded (from 17.7% to 13.6%, *P* = 0.0007), but all other IFDs remained stable (Fig. 1C).

The means of diagnosis differed according to IFDs and underlying conditions (Table 1 and 2) and evolved over time (Fig. 1D and E and below). PCR-positive samples were mostly respiratory (2,015/2,359, 85.4%) and blood (*n* = 273, 11.6%) samples.

Global mortality at 3 months was 38.8% (3,364/8,669 patients for whom the information was available) with significant variations according to the IFD diagnosed (see below) and the main underlying condition. The global mortality rate did not change over time nor did that of each IFD (Fig. 1F).

### Major IFDs and trends over time.

### Yeast fungemia.

Yeast fungemia (5,444 episodes) was recorded in 5,222 patients, namely, mostly adults with a median age of 63.8 years (M:F ratio, 1.6:1 in adults and 1.2:1 in children). The main underlying conditions were malignancies (37.8%, including 1,137 solid tumors, 786 HM, and 49 both), recent surgery (31.1%), and stay in ICU (35.8%) with a trend toward fewer patients with recent surgery (38.9% to 35.7%, *P* = 0.00279) or a stay in ICU (37.8% to 32.0%, *P* = 0.0056), more elderly (11.3% to 14.4%, *P* = 0.0388), and more patients with type 2 diabetes (10.9% to 15.1%, *P* = 0.0197) over time. Global mortality rate was 36.3% (1,640/4,521) and 47.8% (2,082/4,354) at 1 and 3 months, with no change over time.

The species were common ascomycetes (*n* = 4,875, 89.6%), including 2,659/4,875 C. albicans (54.5%), 900 Candida glabrata (18.5%), 664 Candida parapsilosis (13.6%), 400 Candida tropicalis (8.2%), 164 Candida krusei (3.4%), and 88 Candida kefyr (1.8%) with stable proportions over time. Echinocandin resistance excluding C. parapsilosis remained low (2.7%, 22/828 of common species). Rare species included rare ascomycetes (*n* = 401, 7.4%) and basidiomycetes (*n* = 168 [3.1%] including 97 C. neoformans). Mixed species were detected in 235 cases (4.3%). Concurrent IFDs were diagnosed in 74 cases (including 53 IAs, 7 mucormycoses, and 9 PJPs). Overall, 358 patients (6.9%) experienced more than 1 IFD. Echinocandin prescription increased over time (46.7% to 61.8%), while that of fluconazole decreased (30.4% to 19.4%, *P* < 0.0001). The proportion of children, major underlying conditions, prior exposure to antifungal drugs, and global mortality rate at 3 months differed significantly among the four categories (common *Candida* species, uncommon ascomycetous yeasts, Cryptococcus neoformans, and other basidiomycetous yeasts) (see Table S2 in the supplemental material).

10.1128/mbio.00920-22.3 TABLE S2 Characteristics of the 5,444 episodes of yeasts fungemia in 5,222 patients (RESSIF network, 2012 to 2018, France). Download Table S2, PDF file, 0.03 MB. Copyright © 2022 Bretagne et al. 2022 Bretagne et al. https://creativecommons.org/licenses/by/4.0/ This content is distributed under the terms of the Creative Commons Attribution 4.0 International license.

### Invasive aspergillosis.

IA (1,661 episodes) was mostly diagnosed in adults (1,588/1,638) with a median age of 59.3 years (M:F ratio, 1.8:1 in adults and 1.1:1 in children, *P* = 0.046). Hematological malignancies were diagnosed in 65.8% (*n* = 1,078). Underlying conditions were divided into nine exclusive hierarchical categories (22) (see Table S3 in the supplemental material), as follows: acute leukemia without peripheral stem cell or bone marrow allograft, allograft, lymphoma, SOT, solid tumors, other HM, chronic inflammatory diseases, respiratory diseases, and others, with no evolution over time. Global mortality associated with IA was 34.5% (526/1,526) and 42.5% (652/1,536) at 6 weeks and 3 months, respectively, with no change over time. For 157 patients (9.6%), IA was not the first IFD recorded. Overall, 296 patients (18.1%) experienced more than one IFD.

10.1128/mbio.00920-22.4 TABLE S3 Characteristics of the 1,661 episodes of invasive aspergillosis in 1,638 patients (RESSIF network, 2012 to 2018, France). Download Table S3, PDF file, 0.04 MB. Copyright © 2022 Bretagne et al. 2022 Bretagne et al. https://creativecommons.org/licenses/by/4.0/ This content is distributed under the terms of the Creative Commons Attribution 4.0 International license.

Among the 1,661 episodes, 46 (2.7%) were due to mixed Aspergillus species. In addition, 184 (11.1%) concurrent infections were diagnosed in the same anatomical site in 130 (54 mucormycoses, 49 PJPs, 13 fusarioses, and 14 others) or in another body site in 60 (57 fungemia, 5 PJPs, and 6 other IFDs) cases. There was a significant trend toward more concurrent infections over time (7.1% to 14.6%, *P* = 0.0060). Global mortality at 6 weeks was significantly higher in the case of concurrent infections (92/168, 54.8%) than in their absence (434/1,358, 32.0%; *P* < 0.0001). It was significantly higher with fungemia (41/51, 80.4%) or mucormycosis (27/49, 55.1%) than PJP (18/43, 41.9%) or other IFDs (22/62, 35.5%) (*P* < 0.0001).

**FIG 3:**
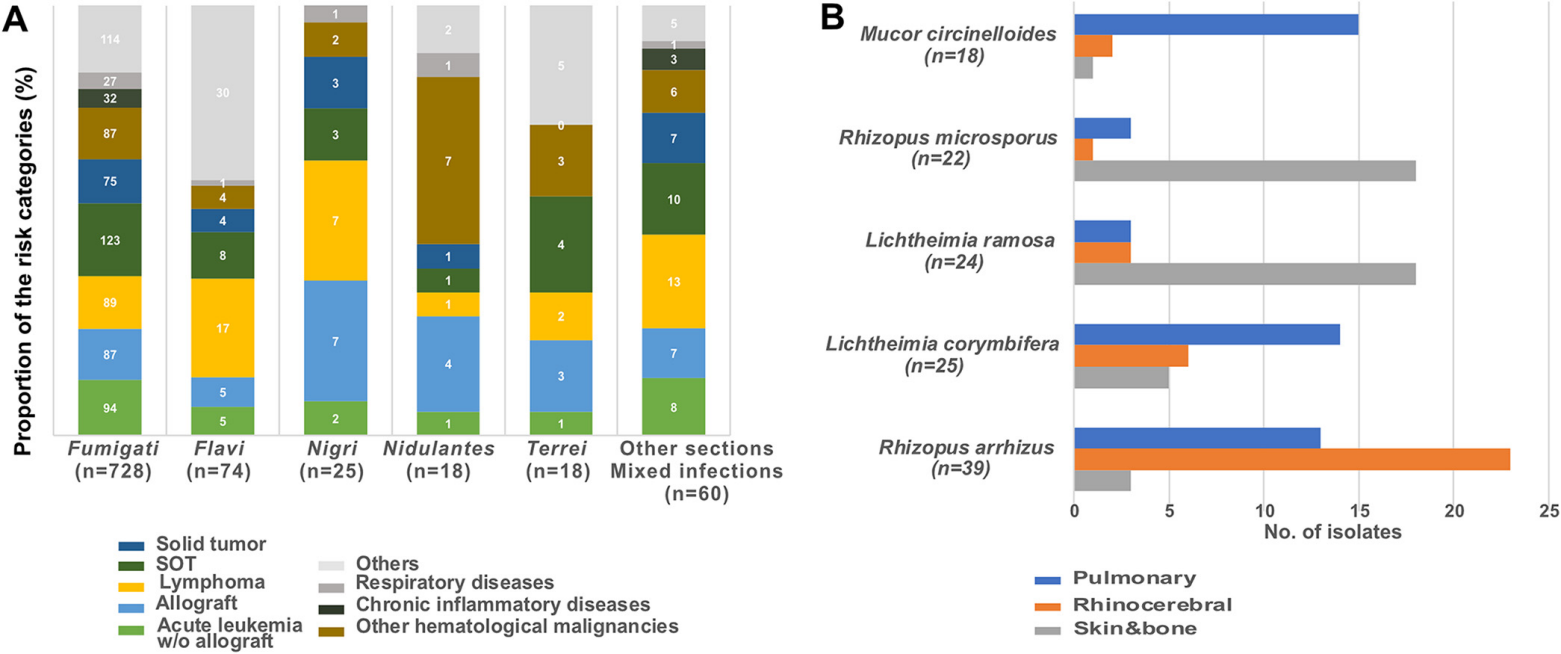
Specific host and species patterns (RESSIF network, France, 2012 to 2018). (A) Distribution of the underlying conditions for the 845 patients for whom the diagnosis of proven or probable invasive aspergillosis was associated with culture of Aspergillus spp. belonging to various sections. (B) Distribution of pulmonary, rhino cerebral, and skin/bone infections according to the species of *Mucorales* identified after culture of the specimens.

The diagnostic procedures did not change over time except for PCR-based methods which use rose from 14.3% to 30.2% (*P* < 0.0001) (Fig. 1F). Most of the isolates recovered were from the *Fumigati* section with no change over time. Antifungal susceptibility was assessed only upon request for A. fumigatus. Of the 771 isolates recovered, 16 (2.1%) had an itraconazole MIC of >1 mg/L associated with a *CYP51A* mutation and tandem repeat (TR) in the gene promoter region (TR34/L98H [*n* = 13]; TR46/Y121F/T289A, F46Y/M172V/E427K, and wild type [*n* = 1 each]). Initial antifungal treatment was mainly voriconazole (1,045/1,613, 64.8%). Significant differences regarding age, proportion of children, pre-exposure to antifungals, stay in ICU, body sites involvement other than lungs, diagnostic procedures, species involved, treatment, and global mortality were observed according to the underlying condition (Table S3, Fig. 3A).

### Mucormycosis.

Mucormycosis was diagnosed mostly in adults (300/313 patients, 95.1%) with a median age of 57.5 (M:F ratio, 1.9:1 in adults and 1.2:1 in children). Patients were assigned the following exclusive categories of risk factors: HM (62.6%), SOT (7.7%), diabetes mellitus (8.6%), skin injury (14.4%), and others (6.7%) (see Table S4 in the supplemental material). Global mortality was 46.1% (137/297) at 1 month and 59.0% (177/300) at 3 months. For 19.2% of the patients (*n* = 60), mucormycosis was not the first IFD recorded. Overall, 104 patients (33.2%) experienced more than 1 IFD.

10.1128/mbio.00920-22.5 TABLE S4 Characteristics of the 343 episodes of mucormycosis in 343 patients (RESSIF network, 2012 to 2018, France). Download Table S4, PDF file, 0.03 MB. Copyright © 2022 Bretagne et al. 2022 Bretagne et al. https://creativecommons.org/licenses/by/4.0/ This content is distributed under the terms of the Creative Commons Attribution 4.0 International license.

Concurrent IFDs were diagnosed in 73 cases (56 IA, 7 fungemia, 7 fusarioses, 1 PJP, and 9 other IFDs) and mixed *Mucorales* species in 2 cases. There was a trend toward more concurrent infections over time, which was significant for those in the same anatomical site (19.4% to 35.9%, *P* = 0.0371). Global mortality rate was not altered by concurrent infection (not shown). Positive PCR result in serum was the only means of diagnosis in 55 cases (17.5%) mostly in HM patients (see Table S5 in the supplemental material). PCR-based techniques were used increasingly over time (33.3% to 92.3%, *P* < 0.0001) (Fig. 1F). The following six species were responsible for 85% (173/203) of the episodes diagnosed with positive culture: Rhizopus arrhizus (*n* = 41, 23.7%), Lichtheimia corymbifera (*n* = 30, 17.3%), *Rhizomucor pusillus*, Mucor circinelloides, Lichtheimia ramosa (*n* = 27, 15.6% each), and Rhizopus microsporus (*n* = 21, 12.1%). Species distribution varied with body localization (Fig. 3B) (*P* < 0.0001).

10.1128/mbio.00920-22.6 TABLE S5 Characteristics of the 2,106 episodes of Pneumocystis jirovecii pneumonia in 2,087 patients (RESSIF network, 2012 to 2018, France). Download Table S5, PDF file, 0.03 MB. Copyright © 2022 Bretagne et al. 2022 Bretagne et al. https://creativecommons.org/licenses/by/4.0/ This content is distributed under the terms of the Creative Commons Attribution 4.0 International license.

### Pneumocystis jirovecii pneumonia.

PJP (*n* = 2,106) was diagnosed mostly in adults (2,047/2,087 patients, 98.1%) with a median age of 61.7 years (M:F ratio, 1.8:1 in adults and 1.7:1 in the 40 children). The patients were assigned to the following exclusive categories: HIV infection (19.3%), SOT (15.0%), malignancies (46.8%), and others (36.0%). Malignancies consisted in 609 HM (including 43.7% acute leukemia, 20.9% lymphoma) and 458 solid tumors (including lung in 34.3%, digestive or genital tract in 10.0% each). This others group included various, potentially combined, conditions, as follows: corticosteroids (*n* = 228, 57.9%), autoimmune disorders (*n* = 218, 55.3%), stay in ICU (*n* = 125, 31.7%), type 2 diabetes (*n* = 66; 16.8%), liver cirrhosis (*n* = 46, 11.7%), and chronic respiratory diseases (*n* = 51, 12.9%). Global mortality was 18.7% (351/1,873) and 26.3% (478/1,819) at 1 and 3 months, respectively, and was significantly higher in HIV-negative than that in HIV-positive patients (332/1519, 21.9% versus 19/354, 5.4%; and 451/1468, 30.7% versus 27/351, 7.7% at 1 and 3 months, respectively; *P* < 0.0001) (Table S5). PJP was not the first IFD recorded for 2.3% of the patients (*n* = 42). Overall, 162 of the patients (7.8%) experienced more than 1 IFD, including a recurrence of PJP in 19 during the survey.

Concurrent IFDs were diagnosed in 74 cases (including 54 IAs, 13 cryptococcoses, and 9 fungemia). They occurred more often in the same anatomical site in patients with malignancy than in the other groups (*P* = 0.037). Global mortality at 1 month was higher in patients with (21/66, 31.8%) than without (330/1,807, 18.3%; *P* = 0.006) concurrent infections. The proportion of proven cases was 74.3% (304/409) and 37.9% (643/1,697) among HIV-positive and -negative patients, respectively (*P* < 0.0001). The means of diagnosis were used unevenly in the four groups (Table S5). The trends in the use of diagnostic tools were oriented inversely with microscopy decreasing, while PCR-based diagnosis increased (*P* < 0.0001) (Fig. 1E and F). Only 5.7% (*n* = 119) of the patients received PCP prophylaxis. Cotrimoxazole was used for treatment in 86.8% of the cases.

## DISCUSSION

We showed here the potential of a university hospital’s network based on active and perennial collaboration with well-trained mycologists and infectious disease clinicians for surveying IFDs. The IFDs recorded through RESSIF include fungemia but also more challenging diagnoses, such as invasive mold infections or PJP. Of note, each case was reviewed carefully at the NRCMA based on European Organization for Research and Treatment of Cancer and the National Institute of Allergy and Infectious Diseases Mycoses Study Group (EORTC/MSG) criteria, checking discrepant/missing information, and characterizing all isolates of rare species or of uncommon phenotype. Even if medical specialties are not distributed evenly across centers altering the distribution of populations at risk of IFDs, we can reasonably expect that the sustained surveillance and the aggregation of data collected in 29 academic centers totaling almost 11,000 cases smoothened the center and even geographical effects.

The design of the RESSIF network allowed us to describe the landscape of IFDs in France between 2012 and 2018 and their own dynamics. IFDs consisted mainly of fungemia (49%), PJP (20%), and IA (19%) confirming the previous ranking in France (15), but mucormycosis is now more often diagnosed than cryptococcosis. Despite the availability of new diagnostic procedures and publications of guidelines (23, 24), the global 3-month mortality remained unchanged over the 7 years, as recently shown for yeast fungemia (25). Overall, the global incidence was 2.21 IFDs/10,000 hospitalization days, with a significant increase over time (Fig. 1A). This increase paralleled the French policy to reduce hospital beds. This reduction may have contributed to an increase in the proportion of more fragile/susceptible patients in hospitals, as shown before (15). This increase is probably linked to the global aging of the French population, as observed in RESSIF, with an increased proportion of patients with diabetes or autoimmune disorders and of elderly patients.

The increase in IFD incidence was supported mainly by an increase in fungemia following a similar observation at the national (2001 to 2010) (15) or regional (Paris area, 2010 to 2017) (18) levels. There is no microbiological explanation for it and no dramatic change in the species diagnosed or their proportions even if preexposure to antifungals influenced the species recovered as previously shown (18, 19, 21). The population diagnosed with yeast fungemia changed over time with more elderly, more patients with type 2 diabetes, and fewer patients with recent surgery or ICU stay. However, the proportion of patients with malignancies remained stable. As shown in the Paris area (18), antifungal prescriptions evolved with more echinocandins and less fluconazole, but the proportion of resistant isolates did not change (26). Global mortality rate associated with yeast fungemia was 47.8% at 3 months. Whether this result is related to more fragile patients dying with their IFD rather than from the IFD remains a major question.

In contrast to fungemia, there was a significant decrease in the proportion of IA (from 17.7% to 13.6%), without a significant change in its incidence (from 0.38 to 0.32). Underlying conditions remained stable over time, including HM, age, and ICU stay, as well as the species involved that differed according to the risk factors. Azole resistance remained anecdotal (2.1%) for A. fumigatus isolates recovered in patients with IA, being different from chronically colonized patients (27). The use of PCR-based diagnosis was still limited and depended on local practice and population at risk. Global mortality (34.5% and 42.5% at 6 weeks and 3 months, respectively) was in line with previous reports (15) and unfortunately did not change over time. Concurrent fungal infections were recorded in 11.1% of the cases. Similar to findings by Danion et al., who showed the deleterious effect of coinfections during IA (28), global mortality associated with IA at 6 weeks (34.5%%) was much higher for those diagnosed with concurrent fungemia or mucormycosis, whereas PJP or other IFDs had no impact. The increased proportion of coinfections over time could explain the lack of decrease in global mortality despite advances in the management of IA. The relative decrease in the incidence of IA may result from more efficient antifungal prophylaxis in high-risk patients (replacement of the posaconazole solution by tablets providing better absorption [29]). An in-depth analysis is requested to better analyze the factors contributing to these trends. We also acknowledge that IA associated with chronic respiratory diseases was certainly underreported for a lack of clear-cut criteria. It is unlikely to have decreased over time given the high proportion of smokers in France (32% of the population [30]).

Compared with the previous report (15), the data recorded through RESSIF showed a nonsignificant increased proportion of mucormycosis without significant trends in terms of incidence or global mortality over time. The 3-month mortality recorded here (59%) is higher than that in the 2005 to 2007 RetroZygo study (44%) (31), possibly resulting from a higher proportion of patients with malignancies who have the worse prognosis (50% in RetroZygo, 62.3% here). The cases diagnosed in 2000 to 2010 and now are probably different by the underlying diseases themselves with more severely ill patients. This difference may also be a paradoxical consequence of diagnosis by PCR rather than culture in these patients. Indeed, the lack of prognosis improvement contrasts with major changes in the tools used for the diagnosis of mucormycosis with the advent of PCR-based methods in routine laboratory practice (32) and the decreased use of culture. The reason why an earlier diagnosis, as provided by the *Mucorales* PCR, would not impact global mortality is not trivial and will deserve further analysis, keeping in mind that up to 2017, the PCR test was only confirmatory and that some tests are outsourced for a lack of local resources, thus limiting the benefit of an earlier diagnosis.

The incidence of PJP remained stable over the study period. Although PJP has been associated mainly with HIV infection, it is not the case anymore (33). The HIV-infected patients accounted for only 19.3% of the patients diagnosed with PJP. The diagnosis relies nowadays more on PCR than on microscopy even though the significance of microscopy-negative and PCR-positive samples is sometimes questioned by clinicians, especially in HIV-negative patients. Indeed, the fungal load is lower in these patients (34), as shown here by a higher proportion of probable PJP in HIV-negative patients. However, the quantification as inferred by PCR assays was not considered here for a lack of consensus on threshold and method (35). Importantly, global mortality associated with PJP was significantly higher in HIV-negative than in HIV-positive patients, suggesting that even low *P. jirovecii* DNA loads should not be neglected (36).

Using the RESSIF data to extrapolate incidences at a national scale is challenging. Since RESSIF encompassed 45% of national capacity of university hospitals in terms of hospitalization days, it could be done for IA, mucormycoses, and PJP; these IFDs occur mainly in immunocompromised patients mostly treated in university hospitals. Fungemia however can occur in nonimmunocompromised patients in hospitals not included in the RESSIF network for a lack of a referent mycologist. According to the administrative data for France, a mean of 1,556 candidemia/year was recorded between 2001 and 2010, leading to an annual incidence of candidemia of 2.5 to 3.5/100,000 population/year (15). Here, we collected in average 737 cases/year suggesting that we missed at least 53% of the candidemia knowing that indeed the figures are probably higher with the increase of the French population and its aging. Overall, it is likely that the IFDs burden in France is more than twice what we recorded in RESSIF since we also excluded IFDs following surgery, such as fungal peritonitis (37), and those for which clear criteria are still missing, such as in patients with chronic respiratory diseases.

Even though RESSIF was designed to overcome limitations of surveillance programs based on administrative data, it still has some, as follows: (i) it does not cover the entire country (see above with the estimations at a national level) as opposed, for example, to the Danish survey on candidemia (8); (ii) despite providing clinical data which are usually missing in other surveillance systems, global and attributable mortality analysis would have required additional information, such as preemptive and prophylactic therapies, therapeutic follow-up, and severity grading for ICU patients, which cannot be collected reliably unless specific studies are set up; and (iii) the electronic case report form (e-CRF) includes predefined variables potentially preventing the recording of new events. Interactions with our colleagues and SPF permit uncovering such cases. Altogether the e-CRF flexibility and interactions with SPF allowed to rapidly evaluate the Saprochaete clavata outbreak in 2012 (38), to assess the small number of Candida auris in France (12), or more recently to investigate the burden of IFDs in the context of COVID-19 (39). The e-CRF is indeed easily amendable through the VOOZANOO platform to record new parameters (e.g., COVID-19 context). (iv) The last issue concerns the completeness of the recordings for a lack of a double check with the hospital databases. We regularly assessed the reporting evolution for each CC-NCRMA and challenged suspicious annual variation. However, to consider the possible weariness of some participants that could lead to incomplete reporting in the future, we are planning to reduce the e-CRF.

We are convinced that implementing this program and involving clinicians and mycologists in the recording helped increasing the awareness on IFDs both at the government agency and the hospital levels. Increasing the knowledge on the epidemiology of IFDs may in turn improve patient prognosis by triggering new prophylactic measures or targeted diagnostic tests for specific populations. RESSIF is already an invaluable tool to use to monitor IFDs and their evolution in France and to allow for an in-depth analysis of unusual settings, including rare pathogens (40) or specific conditions (e.g., diabetes, cirrhosis, and elderly). These analyses could help define new diagnostic or therapeutic strategies and contribute to a better knowledge of IFDs. They may also pave the way to new hypotheses on IFD pathogenesis and new avenues for investigating host/pathogen interactions.

Another benefit of data centralization is the update of taxonomic names based on the NRCMA expertise. While such a change rarely affects therapeutic management, it is important for studies on the relationship between genera/species/clinical presentation and/or outcome for specific groups of pathogenic fungi. In that regard, a request through Institut Pasteur FungiBank could trigger collaborations.

## MATERIALS AND METHODS

### Network.

RESSIF is based on active, sustained, and voluntary participation of collaborative centers (CC-NRCMA; *n* = 13 in 2012, 29 later on, and 21 active between 2013 and 2018). These CC-NRCMAs correspond to 21/32 university hospitals totaling approximately 45% of hospitalization days of all French university hospitals and covering 15/18 French regions, including overseas territories (see Fig. S1 in the supplemental material). Each CC-NRCMA aggregates several hospitals/wards dealing with adult and pediatric patients with various underlying conditions. A referent well-trained medical mycologist is responsible for the accuracy and completeness of the records based on local diagnosis in collaboration with clinicians. A signed agreement between the CC-NRCMA and the NRCMA determined the duration of the collaboration, the duties, and the benefits for each party. Participating centers and individuals had no financial reward.

10.1128/mbio.00920-22.1 FIG S1 Maps of France showing the RESSIF network (2012 to 2018). (A) Distribution of the centers (1 to 4 centers/region; median, 2) with 13/14 regions of France involved. (B) The number of cases of invasive fungal diseases reported for each region is indicated on each sphere. Download FIG S1, TIF file, 0.7 MB. Copyright © 2022 Bretagne et al. 2022 Bretagne et al. https://creativecommons.org/licenses/by/4.0/ This content is distributed under the terms of the Creative Commons Attribution 4.0 International license.

### Information system VOOZANOO and database organization.

The real-time Web-based electronic case report form (e-CRF) was developed using VOOZANOO, an open-source platform to rapidly create questionnaires and surveillance applications (http://www2.voozanoo.net/). The database is saved daily on a server approved for medical data storage. The architecture is hierarchical allowing the administrator (NRCMA) to define levels (NRCMA/city/hospital/ward), and roles with specific rights (referent mycologist and collaborator/clinician) for each account. The rights concern the files (creation, update, deletion, and download) and the tools (creation or simple use of queries and variables available for comparison). Each CC-NRCMA has full access to its own data, is free to perform any in-house study, and can request access to part of the whole data set (32). The coordinating committee, including representatives of CC-NRCMA and NRCMA, oversees validation of the projects. An annual meeting held at Institut Pasteur allows representatives to share updates on the network and results of substudies.

### Questionnaire.

Data on the patient (gender, date of birth, ward, underlying conditions, immunosuppressive drugs, foreign devices, and travel history) and on the episode of IFD (signs, symptoms, imaging, means of diagnosis, direct examination, culture, histology, PCR, antigen detection, specimens studied, prior antifungal prescription, and coinfections), initial therapy within the first 48 hours (antifungals and surgery), and outcome at 3 months (dead, alive, or lost) with date of last news/death were recorded. Most variables were categorical with unique or multiple choices or drop-down menus to facilitate secure recording and analysis. Some free text entries allowed one to add relevant information. Missing or ambiguous information for outstanding data and failure to send the isolate triggered queries (through e-CRF, mail, or phone call). All edits were traced. A unique “case identifier” allowed one to link episodes for a given patient.

### Strain identification.

The CC-NRCMAs are responsible for fungal identification. Nevertheless, they agreed to send their isolates for central characterization at the NRCMA, except for the common species (Candida albicans, Candida glabrata, Candida tropicalis, Candida parapsilosis, Candida krusei, Candida kefyr, and Aspergillus fumigatus), unless the isolate exhibited an unusual antifungal susceptibility profile.

The NRCMA provided polyphasic identification based on morphology, matrix-assisted laser desorption ionization–time of flight (MALDI-TOF; Bruker biotyper, Germany) mass spectrometry, DNA barcoding (41–43), and antifungal susceptibility testing based on the EUCAST broth microdilution method (42, 44). Results of the final identification and MICs were mailed individually and recorded in the e-CRF. In case of discrepancy for fungal identification, that achieved by the NRCMA was used for the analysis. DNA sequences and MIC values were also made available through Institut Pasteur FungiBank. Aggregated yearly updated data are available online (https://www.pasteur.fr/fr/file/39845/download).

### Data analysis.

Each record was assigned to one of the six main exclusive categories of risk for IFD (using the following hierarchy: malignancy, solid organ transplantation [SOT], recent surgery [<1 month] excluding curative management of the IFD, HIV infection, and other conditions known as risk factors for specific IFDs [e.g., diabetes, corticosteroid therapy, and systemic inflammatory disease]) or none known. Complex cases were reviewed by two NRCMA staff members.

IFDs were recorded as invasive aspergillosis (IA), PJP, mucormycosis, cryptococcosis, fusariosis, endemic mycosis, fungemia (if not included in the specific IFDs listed), or other IFDs (rare localizations due to common species and infections due uncommon species not listed above). Abscesses following surgery, peritonitis, esophagitis, and pyelonephritis were not recorded. Some patients experienced multiple IFDs that were considered concurrent if within ≤15 days for pulmonary infections and ≤5 days for other IFDs or subsequent otherwise. Concurrent coinfections involved different body sites (e.g., pulmonary IA and yeast fungemia) and/or multiple species or genera at the same anatomical site. Recurrence was considered 6 months after mycological cure (10 days for yeast fungemia).

IFDs were classified as proven and probable according to the 2008 European Organization for Research and Treatment of Cancer and the National Institute of Allergy and Infectious Diseases Mycoses Study Group (EORTC/MSG) criteria (45). Possible cases were not analyzed given the high degree of diagnostic uncertainty (45). IA cases diagnosed only by positive PCR tests were included, anticipating the new EORTC/MSGERC criteria (2). Even if they were not yet approved, we used the same approach for mucormycosis. For PJP, classification relied on microscopy and PCR results, as follows: proven with positive microscopy and probable with negative microscopy and a positive qPCR. Global mortality was assessed 1 and 3 months after diagnosis.

Data were analyzed anonymously using Stata/SE 15.1 for Mac (StataCorp., College Station, TX). To avoid autocorrelation, characteristics of the patients, including death, were analyzed for the first episode recorded for the global analysis and for the first episode of the corresponding IFD for the analysis of specific IFDs. Trends and incidence were calculated for the 21 CC-NRCMA perennials between 2013 and 2018. Incidence rates were calculated per 10,000 hospitalization days using annual hospital activity data (SAE administrative data, Ministry of Health, https://www.sae-diffusion.sante.gouv.fr/sae-diffusion/recherche.htm). Official data on the French population were available online (https://www.insee.fr/fr/statistiques/2381476). Their evolution was analyzed by linear regression. The trends of specific parameters were analyzed using the chi-square test for trends. Comparisons were based on chi-square or Fisher’s exact test when needed for discrete variables.

### Ethical considerations.

The research was carried out in compliance with the French law and the Declaration of Helsinki (as adopted in 2000). The surveillance of the NRCMA was approved by the Institut Pasteur Institutional Review Board 1 (2009–34/IRB) and the “Commission National de l'Informatique et des Libertés” according to the French regulation.
