# Supplementary material for: Active Surveillance Program to Increase Awareness on Invasive Fungal Diseases: the French RESSIF Network (2012 to 2018)
Source: mBio. 2022 May 2;13(3):e00920-22. doi: 10.1128/mbio.00920-22 (PMC9239099; doi:10.1128/mbio.00920-22)
Supplement: TABLE S1 [file mbio.00920-22-st001.pdf]

Table S1: Underlying conditions in 10,154 patients diagnosed with invasive fungal diseases in France (RESSIF, 2013-2018)

	Patients diagnosed with the corresponding underlying condidion		
	total number	% of the total	% of the category
**Malignancies**	**5140**	**50.6%**	
**Haematological malignancies**	**2634**		51.2%
Lymphoma	1166		44.3%
Acute leukemia	863		32.8%
Other	747		28.4%
**Solid tumors**	**2364**		46.0%
Digestive tract	884		37.4%
Genital tract	302		12.8%
Lung	369		15.6%
Urinary tract	246		10.4%
ENT	203		8.6%
Others	502		21.2%
**Surgery**	**2264**	**22.3%**	
Digestive tract	1033		45.6%
Cardiovascular	348		15.4%
Orthopedic	240		10.6%
Various	643		28.4%
**Solid organ transplantation**	**873**	**8.6%**	
Kidney	473		54.2%
Liver	173		19.8%
Heart	116		13.3%
Lung	101		11.6%
Others	10		1.1%
**HIV infection**	**638**	**6.3%**	
**Other or additional underlying conditions**			
**Stay in ICU**	**3104**	**30.6%**	
**Diabetes**	**1444**	**14.2%**	
**Autoimmune disorders**	**627**	**6.2%**	
**Liver cirrhosis**	**424**	**4.2%**	
**Skin injuries/ local trauma**	**268**	**2.6%**	
**Multiple underlying risk factors**	**1271**	**12.5%**	
