# Supplementary material for: Active Surveillance Program to Increase Awareness on Invasive Fungal Diseases: the French RESSIF Network (2012 to 2018)
Source: mBio. 2022 May 2;13(3):e00920-22. doi: 10.1128/mbio.00920-22 (PMC9239099; doi:10.1128/mbio.00920-22)
Supplement: TABLE S2 [file mbio.00920-22-st002.pdf]

Table S2: Characteristics of the 5,444 episodes of yeasts fungemia in 5,222 patients (RESSIF network, 2012-2018, France)

	Common *Candida* species	Uncommon ascomycetous yeasts	*Cryptococcus neoformans*	Other basidiomycetous yeasts	Total	p
**Characteristics of the patients**, n/total (%)						
Male gender	2890 / 4691 (61.6%)	227 / 367 (61.9%)	73 / 96 (76.0%)	41 / 68 (60.3%)	3231 / 5222 (61.9%)	0.031
Children (<15-year)	224 / 4691 (4.8%)	35 / 367 (9.5%)	0 / 96 (0.0%)	14 / 68 (20.6%)	273 / 5222 (5.2%)	<0.0001
Median age (IQR)	63.7 (22.6)	58.9 (24.2)	50.15 (26.4)	52.3 (45.15)	63.1 (23)	0.0001
Main predisposing condition						
Malignancy	1763 / 4691 (37.6%)	154 / 367 (42.0%)	27 / 96 (28.1%)	28 / 68 (41.2%)	1972 / 5222 (37.8%)	<0.0001
Recent surgery	1508 / 4691 (32.1%)	101 / 367 (27.5%)	0 / 96 (0.0%)	14 / 68 (20.6%)	1623 / 5222 (31.1%)	
Solid organ transplantation	152 / 4691 (3.2%)	13 / 367 (3.5%)	15 / 96 (15.6%)	0 / 68 (0.0%)	180 / 5222 (3.4%)	
HIV infection	11 / 4691 (0.2%)	2 / 367 (0.5%)	38 / 96 (39.6%)	0 / 68 (0.0%)	51 / 5222 (1.0%)	
Other predisposing condition	1245 / 4691 (26.5%)	95 / 367 (25.9%)	13 / 96 (13.5%)	26 / 68 (38.2%)	1379 / 5222 (26.4%)	
None identified	12 / 4691 (0.3%)	2 / 367 (0.5%)	3 / 96 (3.1%)	0 / 68 (0.0%)	17 / 5222 (0.3%)	
Experience of multiple IFIs (simultaneous or not)	300 / 4691 (6.4%)	41 / 367 (11.2%)	10 / 96 (10.4%)	7 / 68 (10.3%)	358 / 5222 (6.9%)	0.001
**Characteristics of the episodes**, n/total (%)						
Intensive care unit	1751 / 4875 (0.0%)	142 / 401 (0.0%)	18 / 97 (0.0%)	23 / 71 (0.0%)	1934 / 5444 (0.0%)	0.005
Central venous catheter	3375 / 4875 (69.2%)	288 / 401 (71.8%)	17 / 97 (17.5%)	52 / 71 (73.2%)	3732 / 5444 (68.6%)	<0.0001
Prior exposure to antifungal drugs	565 / 4731 (11.9%)	82 / 387 (21.2%)	7 / 93 (7.5%)	20 / 70 (28.6%)	674 / 5281 (7.6%)	<0.0001
Echinocandines	202 / 4731 (4.3%)	39 / 387 (10.1%)	2 / 93 (2.2%)	12 / 70 (17.1%)	255 / 5281 (4.8%)	<0.0001
Azoles	352 / 4731 (7.4%)	40 / 387 (10.3%)	2 / 93 (2.2%)	7 / 70 (10.0%)	401 / 5281 (7.6%)	0.032
Mixed species	186 / 4875 (3.8%)	47 / 401 (11.7%)	0 / 97 (0.0%)	2 / 71 (2.8%)	235 / 5444 (4.3%)	<0.0001
Recurrence	128 / 4875 (2.6%)	19 / 401 (4.7%)	3 / 97 (3.1%)	0 / 71 (0.0%)	150 / 5444 (2.8%)	0.041
Initial antifungal treatment for cases with results of positive culture known before death						
Echinocandin	2119 / 4437 (47.8%)	175 / 377 (46.4%)	0 / 89 (0.0%)	14 / 68 (20.6%)	2308 / 4971 (46.4%)	<0.0001
Azole	1291 / 4437 (29.1%)	83 / 377 (22.0%)	9 / 89 (10.1%)	5 / 68 (7.4%)	1388 / 4971 (27.9%)	
Others including combination	786 / 4437 (17.7%)	104 / 377 (27.6%)	78 / 89 (87.6%)	43 / 68 (63.2%)	1011 / 4971 (20.3%)	
No drug prescribed	241 / 4437 (5.4%)	15 / 377 (4.0%)	2 / 89 (2.2%)	6 / 68 (8.8%)	264 / 4971 (5.3%)	
**Global mortality**, n/total available (%)						
At 1 month	1466 / 3984 (36.8%)	138 / 392 (35.2%)	23 / 86 (26.7%)	13 / 59 (22.0%)	1640 / 4521 (36.3%)	0.019
At 3 months	1867 / 3830 (48.7%)	171 / 387 (44.2%)	25 / 82 (30.5%)	19 / 55 (34.5%)	2082 / 4354 (34.5%)	<0.001
