# Supplementary material for: Active Surveillance Program to Increase Awareness on Invasive Fungal Diseases: the French RESSIF Network (2012 to 2018)
Source: mBio. 2022 May 2;13(3):e00920-22. doi: 10.1128/mbio.00920-22 (PMC9239099; doi:10.1128/mbio.00920-22)
Supplement: TABLE S3 [file mbio.00920-22-st003.pdf]

Table S3: Characteristics of the 1,661 episodes of invasive aspergillosis in 1,638 patients (RESSIF network, 2012-2018, France)

	Acute leukemia w/o allograft	Allograft (Peripheral Stem Cells/marrow)	Lymphoma	Solid organ transplantation	Solid tumor	Other hematological malignancies	Chronic inflammatory diseases	Respiratory diseases	Others	p
**Characteristic of the patients,** n/total (%)										
Male gender	125 / 197 (63.5%)	161 / 283 (56.9%)	256 / 416 (61.5%)	116 / 181 (64.1%)	69 / 113 (61.1%)	133 / 192 (69.3%)	34 / 52 (65.4%)	28 / 38 (73.7%)	116 / 166 (69.9%)	0.088
Children	1 / 197 (0.5%)	13 / 283 (4.6%)	20 / 416 (4.8%)	/	/	8 / 192 (4.2%)	1 / 52 (1.9%)	/	7 / 166 (4.2%)	0.003
Median age (IQR)	62.1 (18.2)	52.1 (24)	60.5 (18.2)	62.1 (22.5)	57.9 (13.9)	64.3 (16.7)	65.9 (20.15)	60.85 (19.15)	65.15 (16.3)	0.0001
Experience of multiple IFIs (simultaneous or not)	43 / 197 (21.8%)	51 / 283 (18.0%)	65 / 416 (15.6%)	41 / 181 (22.7%)	12 / 113 (10.6%)	42 / 192 (21.9%)	9 / 52 (17.3%)	5 / 38 (13.2%)	28 / 166 (16.9%)	0.11
**Characteristics of the episodes**, n/total (%)										
**Stay in intensive care unit**	75 / 199 (37.7%)	40 / 297 (13.5%)	90 / 419 (21.5%)	81 / 184 (44.0%)	51 / 113 (45.1%)	56 / 193 (29.0%)	22 / 52 (42.3%)	31 / 38 (81.6%)	90 / 166 (54.2%)	<0.0001
**Prior exposure to antifungals**	48 / 199 (24.7%)	151 / 297 (53.0%)	158 / 419 (39.5%)	47 / 184 (27.2%)	16 / 113 (14.5%)	55 / 193 (28.6%)	12 / 52 (24.0%)	2 / 38 (5.3%)	22 / 166 (13.8%)	<0.0001
**Main localization**										<0.0001
Lung	182 / 199 (91.5%)	285 / 297 (96.0%)	396 / 419 (94.5%)	157 / 184 (85.3%)	96 / 113 (85.0%)	164 / 193 (85.0%)	44 / 52 (84.6%)	37 / 38 (97.4%)	84 / 166 (50.6%)	
Brain	5 / 199 (2.5%)	2 / 297 (0.7%)	2 / 419 (0.5%)	5 / 184 (2.7%)	2 / 113 (1.8%)	15 / 193 (7.8%)	2 / 52 (3.8%)	/	7 / 166 (4.2%)	
Skin / articulation	4 / 199 (2.0%)	1 / 297 (0.3%)	3 / 419 (0.7%)	4 / 184 (2.2%)	4 / 113 (3.5%)	4 / 193 (2.1%)	1 / 52 (1.9%)	1 / 38 (2.6%)	29 / 166 (17.5%)	
Sinus	2 / 199 (1.0%)	6 / 297 (2.0%)	10 / 419 (2.4%)	10 / 184 (5.4%)	10 / 113 (8.8%)	6 / 193 (3.1%)	4 / 52 (7.7%)	/	26 / 166 (15.7%)	
Others	6 / 199 (3.0%)	3 / 297 (1.0%)	8 / 419 (1.9%)	8 / 184 (4.3%)	1 / 113 (0.9%)	4 / 193 (2.1%)	1 / 52 (1.9%)	/	/	
**Mixed species**	6 / 199 (3.0%)	6 / 297 (2.0%)	7 / 419 (1.7%)	8 / 184 (4.3%)	6 / 113 (5.3%)	5 / 193 (2.6%)	3 / 52 (5.8%)	1 / 38 (2.6%)	4 / 166 (2.4%)	0.360
**Simultaneous (within 5 days) diagnc**	18 / 199 (9.0%)	5 / 297 (1.7%)	10 / 419 (2.4%)	7 / 184 (3.8%)	2 / 113 (1.8%)	9 / 193 (4.7%)	3 / 52 (5.8%)	1 / 38 (2.6%)	5 / 166 (3.0%)	0.002
**Diagnosis means**										
Culture	112 / 199 (56.3%)	126 / 297 (42.4%)	134 / 419 (32.0%)	152 / 184 (82.6%)	90 / 113 (79.6%)	113 / 193 (58.5%)	36 / 52 (69.2%)	32 / 38 (84.2%)	157 / 166 (94.6%)	<0.0001
Microscopy	63 / 199 (31.7%)	73 / 297 (24.6%)	75 / 419 (17.9%)	98 / 184 (53.3%)	48 / 113 (42.5%)	75 / 193 (38.9%)	28 / 52 (53.8%)	15 / 38 (39.5%)	104 / 166 (62.7%)	<0.0001
Antigen	166 / 199 (83.4%)	253 / 297 (85.2%)	348 / 419 (83.1%)	110 / 184 (59.8%)	70 / 113 (61.9%)	141 / 193 (73.1%)	34 / 52 (65.4%)	28 / 38 (73.7%)	75 / 166 (45.2%)	<0.0001
PCR	38 / 199 (19.1%)	59 / 297 (19.9%)	65 / 419 (15.5%)	39 / 184 (21.2%)	16 / 113 (14.2%)	46 / 193 (23.8%)	16 / 52 (30.8%)	6 / 38 (15.8%)	29 / 166 (17.5%)	0.084
**EORTC classification**										
Proven	20 / 199 (10.1%)	19 / 297 (6.4%)	28 / 419 (6.7%)	42 / 184 (22.8%)	21 / 113 (18.6%)	38 / 193 (19.7%)	11 / 52 (21.2%)	6 / 38 (15.8%)	76 / 166 (45.8%)	<0.0001
Probable	177 / 199 (88.9%)	277 / 297 (93.3%)	385 / 419 (91.9%)	142 / 184 (77.2%)	92 / 113 (81.4%)	154 / 193 (79.8%)	40 / 52 (76.9%)	32 / 38 (84.2%)	88 / 166 (53.0%)	
PCR diagnosis	2 / 199 (1.0%)	1 / 297 (0.3%)	6 / 419 (1.4%)	/	/	1 / 193 (0.5%)	1 / 52 (1.9%)	/	2 / 166 (1.2%)	
**Section involved**										
Fumigati	96 / 112 (85.7%)	95 / 126 (75.4%)	92 / 134 (68.7%)	127 / 152 (83.6%)	76 / 90 (84.4%)	89 / 113 (78.8%)	34 / 36 (94.4%)	28 / 31 (90.3%)	118 / 157 (75.2%)	<0.0001
Others	16 / 112 (0.0%)	31 / 126 (0.0%)	42 / 134 (0.0%)	25 / 152 (0.0%)	14 / 90 (0.0%)	24 / 113 (0.0%)	2 / 36 (0.0%)	3 / 31 (0.0%)	39 / 157 (0.0%)	
**Initial antifungal prescribed**, n/n treated (%)										
Voriconazole	136 / 188 (72.3%)	171 / 295 (58.0%)	276 / 411 (67.2%)	105 / 181 (58.0%)	77 / 109 (70.6%)	130 / 187 (69.5%)	36 / 49 (73.5%)	25 / 36 (69.4%)	89 / 157 (56.7%)	<0.0001
Liposomal amphotericin B	22 / 188 (11.7%)	66 / 295 (22.4%)	60 / 411 (14.6%)	33 / 181 (18.2%)	5 / 109 (4.6%)	19 / 187 (10.2%)	3 / 49 (6.1%)	4 / 36 (11.1%)	23 / 157 (14.6%)	
Others including combinations	30 / 188 (16.0%)	58 / 295 (19.7%)	75 / 411 (18.2%)	43 / 181 (23.8%)	27 / 109 (24.8%)	38 / 187 (20.3%)	10 / 49 (20.4%)	7 / 36 (19.4%)	45 / 157 (28.7%)	
**Global mortality**, n/total available (%)										
at 6 weeks	76 / 184 (41.3%)	82 / 273 (30.0%)	100 / 387 (25.8%)	48 / 176 (27.3%)	50 / 102 (49.0%)	67 / 178 (37.6%)	21 / 51 (41.2%)	21 / 34 (61.8%)	61 / 141 (43.3%)	<0.0001
at 3 months	92 / 183 (50.3%)	117 / 280 (41.8%)	130 / 387 (33.6%)	60 / 176 (34.1%)	58 / 102 (56.9%)	83 / 181 (45.9%)	23 / 51 (45.1%)	22 / 35 (62.9%)	67 / 141 (47.5%)	<0.0001
