# Supplementary material for: Active Surveillance Program to Increase Awareness on Invasive Fungal Diseases: the French RESSIF Network (2012 to 2018)
Source: mBio. 2022 May 2;13(3):e00920-22. doi: 10.1128/mbio.00920-22 (PMC9239099; doi:10.1128/mbio.00920-22)
Supplement: TABLE S4 [file mbio.00920-22-st004.pdf]

Table S4: characteristics of the 343 episodes of mucormycosis in 343 patients (RESSIF network, 2012-2018, France)

	Hematological malignancies	Solid organ transplantation	Diabetes	Skin injury	Others	p
**Characteristics of the patients**, n/total (%)						
Male gender	124 / 196 (63.3%)	16 / 24 (66.7%)	22 / 27 (81.5%)	31 / 45 (68.9%)	12 / 21 (57.1%)	0.357
**Children** (<15 years-old)	188 / 196 (95.9%)	23 / 24 (95.8%)	27 / 27 (100.0%)	44 / 45 (97.8%)	18 / 21 (85.7%)	0.135
Median age ± IQR	59 ± 22.0	56.5 ± 12.0	63 ± 17.0	50 ± 37.0	55 ± 25.0	0.1059
**Characteristics of the episodes**, n/total available (%)						
**Prior exposure to antifungals**	116 / 182 (63.7%)	7 / 24 (29.2%)	3 / 24 (12.5%)	7 / 42 (16.7%)	7 / 27 (25.9%)	<0.0001
**Stay in ICU**	38 / 192 (19.8%)	15 / 25 (60.0%)	8 / 25 (32.0%)	30 / 44 (68.2%)	10 / 28 (35.7%)	<0.0001
**Main localization**						
Lung	111 / 192 (57.8%)	15 / 25 (60.0%)	8 / 25 (32.0%)	2 / 44 (4.5%)	8 / 28 (28.6%)	<0.0001
Rhinocerebral	34 / 192 (17.7%)	4 / 25 (16.0%)	13 / 25 (52.0%)	2 / 44 (4.5%)	5 / 28 (17.9%)	
Skin / articulation	13 / 192 (6.8%)	2 / 25 (8.0%)	2 / 25 (8.0%)	38 / 44 (86.4%)	2 / 28 (7.1%)	
Ohers	38 / 192 (19.8%)	3 / 25 (12.0%)	4 / 25 (16.0%)	3 / 44 (6.8%)	6 / 28 (21.4%)	
**Coinfection**	37 / 192 (19.3%)	6 / 25 (24.0%)	2 / 25 (8.0%)	16 / 44 (36.4%)	5 / 28 (17.9%)	0.048
**Diagnosis means**						
Culture	103 / 192 (53.6%)	20 / 25 (80.0%)	19 / 25 (76.0%)	42 / 44 (95.5%)	19 / 28 (67.9%)	<0.0001
Microscopy	106 / 192 (55.2%)	19 / 25 (76.0%)	20 / 25 (80.0%)	44 / 44 (100.0%)	14 / 28 (50.0%)	0.001
PCR	122 / 192 (63.5%)	8 / 25 (32.0%)	12 / 25 (48.0%)	15 / 44 (34.1%)	13 / 28 (46.4%)	0.001
**EORTC classification**						
Proven	81 / 192 (42.2%)	12 / 25 (48.0%)	18 / 25 (72.0%)	21 / 44 (47.7%)	13 / 28 (46.4%)	<0.0001
Probable	62 / 192 (32.3%)	12 / 25 (48.0%)	9 / 25 (36.0%)	24 / 44 (54.5%)	6 / 28 (21.4%)	
PCR only	53 / 192 (27.6%)	0 / 25 (0.0%)	0 / 25 (0.0%)	0 / 44 (0.0%)	2 / 28 (7.1%)	
**Global mortality**, n/total available (%)						
At 1 month	95 / 188 (50.5%)	12 / 23 (52.2%)	10 / 26 (38.5%)	11 / 41 (26.8%)	9 / 19 (47.4%)	0.073
At 3 months	129 / 190 (67.9%)	14 / 24 (58.3%)	11 / 26 (42.3%)	13 / 41 (31.7%)	10 / 19 (52.6%)	<0.0001

* Positive PCR results were obtained mostly from blood samples (48/54 and in 6 cases only from pulmonary samples
