# Supplementary material for: Active Surveillance Program to Increase Awareness on Invasive Fungal Diseases: the French RESSIF Network (2012 to 2018)
Source: mBio. 2022 May 2;13(3):e00920-22. doi: 10.1128/mbio.00920-22 (PMC9239099; doi:10.1128/mbio.00920-22)
Supplement: TABLE S5 [file mbio.00920-22-st005.pdf]

Table S5: Characteristics of the 2,106 episodes of *Pneumocystis jirovecii* pneumonia in 2,087 patients (RESSIF network, 2012-2018, France)

	HIV infection	Solid organ transplantation	Malignancy	Others	Total	p
**Characteristics of the patients**, n/total (%)						
Male gender, n/total (%)	308 / 403 (76.4%)	198 / 313 (63.3%)	621 / 977 (63.6%)	223 / 394 (56.6%)	1350 / 2087 (64.7%)	<0.0001
Children (<15 years-old), n/total (%	1 / 403 (0.2%)	2 / 313 (0.6%)	14 / 977 (1.4%)	23 / 394 (5.8%)	42 / 2087 (2.0%)	<0.0001
Median age ± IQR	47.6 ± 17.2	62.1 ± 16.1	65.3 ± 16.7	66.25 ± 20.0	61.7 ± 20.9	0.0001
Corticosteroid therapy	9 / 403 (2.2%)	139 / 313 (44.4%)	379 / 977 (38.8%)	228 / 394 (57.9%)	759 / 2087 (36.4%)	<0.0001
**Characteristics of the episodes**, n/total available (%)						
**Stay in ICU**	97 / 409 (23.7%)	73 / 317 (23.0%)	269 / 984 (27.3%)	125 / 396 (31.6%)	564 / 2106 (26.8%)	0.029
**Classification of the episode**						
Proven	304 / 409 (74.3%)	172 / 317 (54.3%)	341 / 984 (34.7%)	130 / 396 (32.8%)	947 / 2106 (45.0%)	<0.0001
Probable	105 / 409 (25.7%)	145 / 317 (45.7%)	643 / 984 (65.3%)	266 / 396 (67.2%)	1159 / 2106 (55.0%)	
**Means of diagnosis**						
Microscopy	304 / 409 (74.3%)	172 / 317 (54.3%)	341 / 984 (34.7%)	130 / 396 (32.8%)	947 / 2106 (45.0%)	<0.0001
PCR	302 / 409 (73.8%)	255 / 317 (80.4%)	862 / 984 (87.6%)	367 / 396 (92.7%)	1786 / 2106 (84.8%)	<0.0001
**Antifungal treatment**						
Cotrimoxazole	370 / 400 (92.5%)	276 / 305 (90.5%)	846 / 913 (92.7%)	335 / 357 (93.8%)	1827 / 1975 (92.5%)	0.5930
Atovaquone	24 / 400 (6.0%)	26 / 305 (8.5%)	57 / 913 (6.2%)	17 / 357 (4.8%)	124 / 1975 (6.3%)	
Pentamidine	6 / 400 (1.5%)	3 / 305 (1.0%)	10 / 913 (1.1%)	5 / 357 (1.4%)	24 / 1975 (1.2%)	
**Global mortality**, n/total available (%)						
1 month	19 / 354 (5.4%)	27 / 291 (9.3%)	222 / 870 (25.5%)	83 / 358 (23.2%)	351 / 1873 (18.7%)	<0.0001
3 months	27 / 351 (7.7%)	44 / 283 (15.5%)	303 / 837 (36.2%)	104 / 348 (29.9%)	478 / 1819 (26.3%)	<0.0001
