# Supplementary figures and images for: Active Surveillance Program to Increase Awareness on Invasive Fungal Diseases: the French RESSIF Network (2012 to 2018)
Source: mBio. 2022 May 2;13(3):e00920-22. doi: 10.1128/mbio.00920-22 (PMC9239099; doi:10.1128/mbio.00920-22)

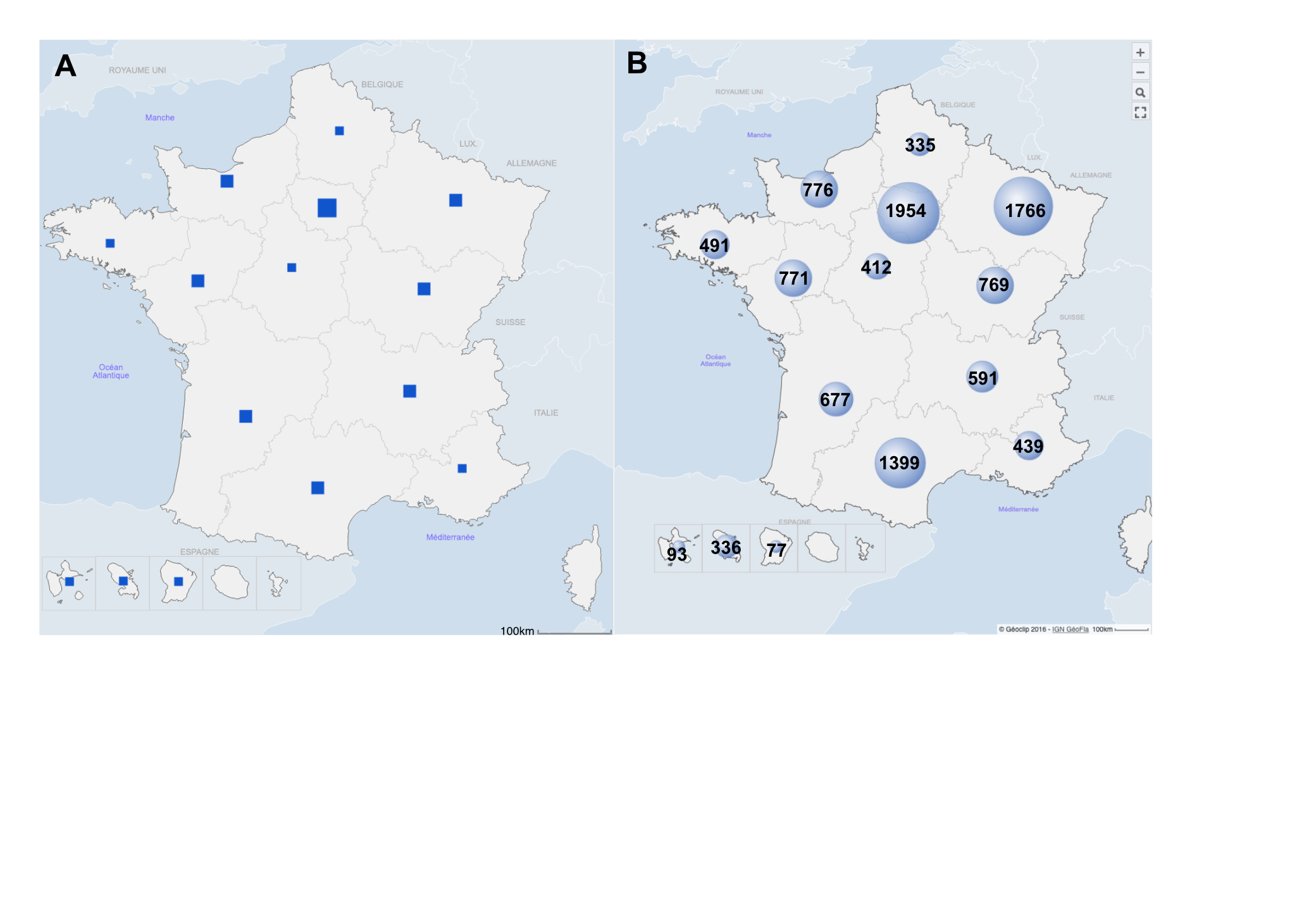

Supplement: FIG S1 [file mbio.00920-22-sf001.tif]
